# Harnessing Technological Advancements for Enhanced Crop Management: A Study on Capsicum Phenology and Automation in Agriculture

**DOI:** 10.12688/f1000research.159518.1

**Published:** 2024-12-12

**Authors:** Deepashri KM, J Satheesh Kumar, Santhosh KV

**Affiliations:** 1 Dayananda Sagar College of Engineering, Department of Electronics and Instrumentation, Visvesvaraya Technological University, Belagavi, Karnataka, India; 2 Manipal Institute of Technology, Department of Instrumentation and Control Engineering, Manipal Academy of Higher Education, Manipal, Karnataka, India

**Keywords:** Crop parameters, Crop phenology, Statistical analysis, t test, ANOVA, Crop management system

## Abstract

**Background:**

Current advancements in communication and information have important impacts on the agricultural sector. Technology has been instrumental in developing innovative approaches to enhancing farming productivity and efficiency while also addressing environmental concerns. With the aid of technology, researchers can collect and analyze vast amounts of agricultural data, enabling a deeper understanding of farming practices and facilitating more informed decision-making through cutting-edge techniques.

**Methods:**

This study focused on the analysis of key agricultural crop parameters, including temperature, humidity, and soil moisture, across various phenological stages of Capsicum cultivation. Statistical hypothesis tests, including t tests and ANOVA, were conducted to identify significant differences in temperature, humidity, and soil moisture across the phenological stages.

**Results:**

The results demonstrated substantial variability in these parameters, emphasizing the importance of tailored crop management strategies.

**Conclusion:**

The insights gained from this statistical analysis can inform the development of autonomous crop management systems that adapt to specific crop needs, thereby enhancing productivity and sustainability in agriculture.

## Introduction

To fulfill the rising need for food, water, and energy, increasing socioeconomic development and population increase, resulting in new problems related to agriculture. To effectively safeguard the sustainability of resources and manage the impact of future agricultural output, rethinking is needed. By employing a combined energy-water-food connection optimization technique, it has been found that it is currently not possible to manage agricultural resources and waste in accordance with agricultural management principles to achieve sustainability. ^1^

The advancement of agricultural modernization is strongly supported by agricultural information. The implementation of modern technological advances to provide relevant and helpful data to users through agricultural information suggestion services has been shown to be a successful remedy with the ongoing evolution of agricultural information infrastructure construction. ^2^ This promoted the creation of autonomous farming systems with a primary focus on irrigation, climate control, and greenhouse monitoring for field work, animal management, and growth control. Effective agricultural production management, which increases both the productivity and safety of agricultural products, can reduce negative environmental effects. Technologies based on sensors are essential in the expanding field of agriculture. Farmers, scientists, and technological manufacturers are working together to develop practical solutions that will increase productivity and result in cost savings. ^3^

Agriculture depends on numerous economic and climatic conditions. The variables that affect agricultural crop production include soil, weather, cultivation, temperature, irrigation, fertilizers, rainfall, harvesting, pesticide and insecticide usage, weeds, and other variables. The study of vital phenomena in plants is known as plant physiology. Understanding different plant biological processes, such as photosynthesis, respiration, transpiration, translocation, nutrient uptake, hormone-regulated plant growth, and other activities that have a significant impact on crop yield, is helpful. ^4^

The profound integration of both contemporary information technology and conventional farming has given rise to the “smart agriculture” era. Smart agriculture addresses automation and intelligence in agriculture. ^5^ Nonetheless, the concerns of statistical security cannot be ignored considering the growth in agriculture facilitated by modern digital technologies. In agriculture, statistical analysis has an important impact on the collection, analysis, and interpretation of numerical data. Agriculturalists can utilize data analytics to develop predictive analytics for future yields, manage resources based on existing trends, and continuously check the health of their crops, ^6^ maximizing profitability and minimizing waste. Statistical analysis of agricultural parameters helps to manage agricultural resource growth by identifying optimal conditions for various crops. Some crops may require a specific range of temperature, humidity, and soil moisture for optimal growth. Understanding the optimal conditions for crop growth, farmers can apply these resources in a targeted manner, reducing waste and maximizing their impact.

## Literature survey

The utilization of IoT devices for monitoring and controlling crop production is prevalent, ^7^ but rural areas often face challenges due to unstable cloud connectivity. Fog computing technology addresses this issue by managing sporadic connectivity and enabling faster data analysis. The experiment employed two datasets: one with air humidity and temperature values and another with soil temperature and moisture values. Fog filters apply unsupervised machine learning techniques to cluster unlabeled data and use supervised learning classification techniques to predict data sample categories.

A comprehensive analysis of 61 selected studies from 221 publications focused on advanced time series models in agriculture. ^8^ Historical data and information techniques for applying time series models are discussed. This review highlighted that deep neural networks enhance simulation and data structure mining capabilities, contributing to end-to-end optimization for environmental prediction. This approach broadens the range of environmental characteristics that agricultural facilities can manage.

A systematic review of agricultural IoT presented the current state, system architecture, and five main IoT technologies. ^9^ Five chosen fields for IoT applications in agriculture were introduced, along with an analysis of the challenges and future growth predictions. The key issues identified include network and interoperability problems, trust, environmental sustainability, and insecurity. An evaluation of IoT technology in agri-food supply chains identified 24 crucial criteria categorized into technological, social, economic, and organizational aspects. ^10^ The DEMATEL approach was used to determine causal relationships, highlighting trust, environmental sustainability, insecurity, interoperability, and network issues as significant factors influencing IoT adoption. These insights can help overcome obstacles in the agri-food sector.

Research on tomato farming in Mayotte aimed to understand production practices and technical choices to promote environmentally friendly practices. ^11^ Field inspections and farmer interviews revealed the overapplication of pesticides and a lack of correlation between application rates and crop health. This study indicated a need for better information and practices related to agricultural health protection.

Data mining techniques such as clustering and regression were used to analyze agricultural data, identifying optimal parameters for enhancing crop production. ^12^ This approach helps improve productivity and climate change resistance by analyzing new and existing data on crops, soil, and climate. An agricultural management system was designed to increase productivity and profitability through effective practices and online product sales. ^13^ Data fusion techniques combine information from various sources for accurate remote findings, aiding intelligent decision-making for resource management based on seasonal and environmental conditions.

The state of modern farm management systems was assessed, highlighting each stage from data collection to decision-making. ^14^ Using AI and robotic technologies, data-driven managed farms can enhance productivity and reduce environmental harm, establishing a foundation for sustainable agriculture.

Agricultural development data from government publications were analyzed using correlation and multiple regression to understand the relationships between cropping intensity and various production performance factors. ^15^ The study revealed significant variance in crop yields influenced by factors such as irrigation and climate conditions. Big Data Analytics was used to construct a weather-based crop prediction system in India. ^16, 17^ Data on various climatic and soil factors were preprocessed and analyzed using the MapReduce framework and k-means clustering. The system provided accurate predictions and was presented through a graphic user interface, aiding agriculturalists in optimizing yields. Systems thinking was implemented to improve agricultural land production by using an internet-based causal loop diagram. ^18^ This approach helps measure variables such as temperature, humidity, and pest activity, addressing issues in planting and pest control.

A comprehensive examination of data mining software in agriculture classified methods for crop production monitoring. ^19^ This study suggested an intelligent crop management system to help farmers make informed decisions based on various environmental and crop parameters. Understanding soil water and salinity variations is crucial for effective irrigation and fertilization management. ^2^ Studies have provided 48-hour estimates of soil salt and water levels, aiding prediction and management in semiarid regions. ^20^ Effective planning and yield assessment are essential in agriculture. The use of data mining techniques for making critical farming decisions, considering commodity prices, soil variance, and environmental factors, was emphasized. Machine learning techniques such as random forest regressor and linear regression were used to analyze agricultural data and optimize crop productivity. ^21, 22^ To address the challenges of climate change and food production, climate-smart farming methods were compared with traditional methods. Irrigation and smart crop management techniques significantly increase yields, demonstrating the potential of data fusion models in optimizing agricultural practices. ^23, 24^

This literature survey highlights the diverse applications of smart agriculture technologies, focusing on IoT, fog computing, data mining, machine learning, and climate-smart farming methods to enhance productivity, sustainability, and resource management in agriculture.

Despite advancements in statistical analysis tools for deciphering crop parameter levels, there remains a significant gap in understanding the inherent variability in crop phenology. Different crops exhibit unique growth stages and respond variably to environmental conditions, posing a challenge in developing generalized models. Research needs to focus on creating more crop-specific models that can accurately capture these unique phenological responses and environmental interactions.

The primary focus of analyzing agricultural parameters is twofold: to assist researchers in expanding their knowledge base and to ensure that farmers benefit from improved crop outcomes and effective field management. Through careful examination of the data, researchers can identify patterns, correlations, and trends, enabling them to make targeted recommendations for optimizing crop production. The vision of designing an autonomous crop management system that is both technologically advanced and flexible remains largely unfulfilled. Existing systems often lack the necessary adaptability to cater to the diverse requirements of different crops and their unique phenologies. Research should focus on developing more flexible, adaptive systems that can tailor interventions and recommendations specific to each crop and its environmental conditions. In conclusion, ongoing advancements in agricultural research, coupled with the application of sophisticated analysis tools, are instrumental in ushering in a new era of precision farming. The goal is to design autonomous systems that not only streamline farming operations but also adapt intelligently to the nuanced needs of different crops, thereby fostering a more sustainable and productive agricultural landscape.

## Method

An agricultural crop management system refers to the combination of various technologies, such as sensors, robotics, and artificial intelligence, to automate and optimize the tasks involved in crop cultivation. Statistical analysis plays a crucial role in agricultural crop management systems. In this work, the *capsicum crop growth* ^25^ experimental dataset was used for the analysis. The dataset consists of agricultural parameters such as temperature, humidity, and soil moisture (at three different levels, SM1, SM2, and SM3) and pressure and light intensity recorded for three months. The Capsicum crop dataset available was recorded during the months of March 2020 (328 samples), April 2020 (2880 samples), May 2020 (2976 samples) and June 2020 (1004 samples) using Libelium hardware. In this work, the temperature, humidity, and soil moisture (SM1) datasets are considered for statistical analysis.

The dataset was analyzed and compared with the available phonological (growth) stage data of Capsicum crops. ^26, 27^ Surveying and consulting agriculturalists revealed that the phenological stages of Capsicum consisted of four growth stages, as shown in Table 1. The data were further divided into four phenological stages.

**Table 1.** Phenological stages of the Capsicum crop.

Growth stage	Stage duration (days)
Vegetative	25
Flowering	10
Fruit set	10
1 ^st^ Harvest	25

**Figure 1.**
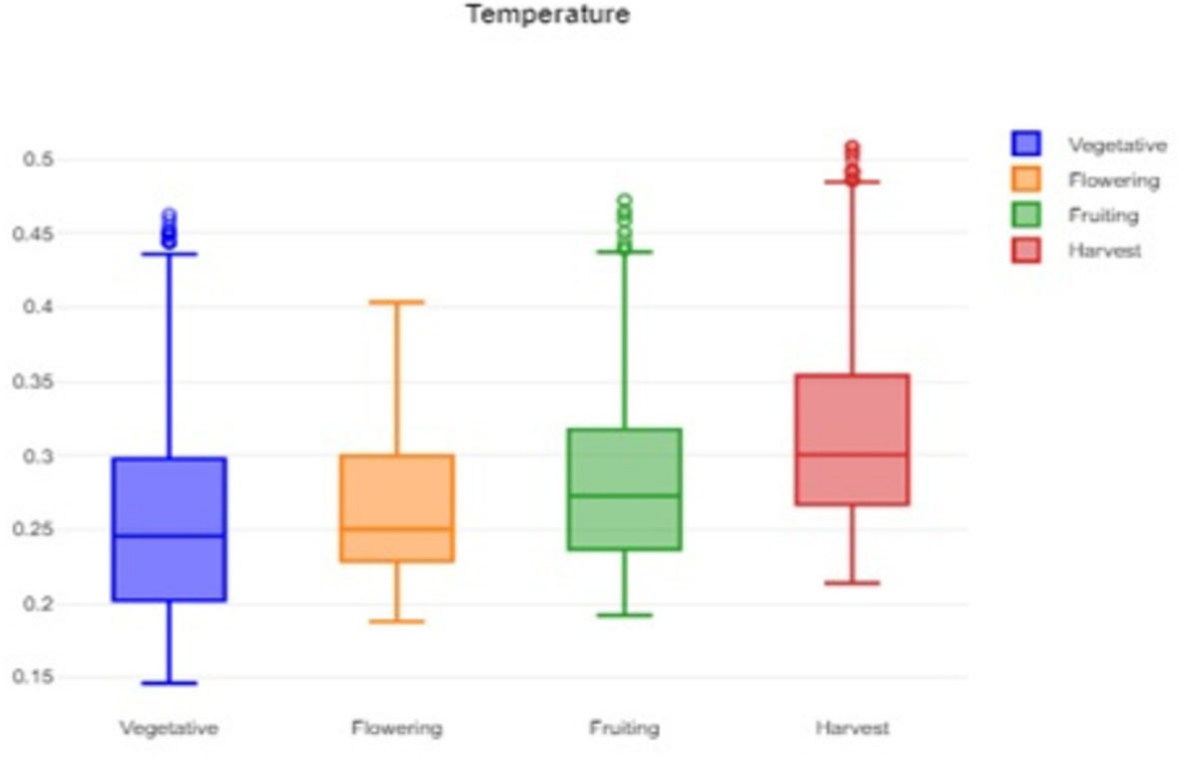
Box plot of the temperature data.

The capsicum seeds are initially seeded in trays or cavities. Seeds germinate approximately one week after sowing. The seedlings in trays are transplanted to fields or planting beds within 30-35 days. ^14^ After transplanting the plants to the vegetative stage followed by flowering, the fruit set and harvest stages of the Capsicum plants were evaluated. The experimental dataset was large, and temperature, humidity, and soil moisture data were available for different growth stages of Capsicum. Box charts are useful visual tools for summarizing and analyzing large datasets. Figure 1 shows that the box plot of temperature data for crop growth stages provides insights into central tendencies, variability, and outliers, which helps to guide decisions related to crop management and identify potential areas for improvement.

**Figure 2.**
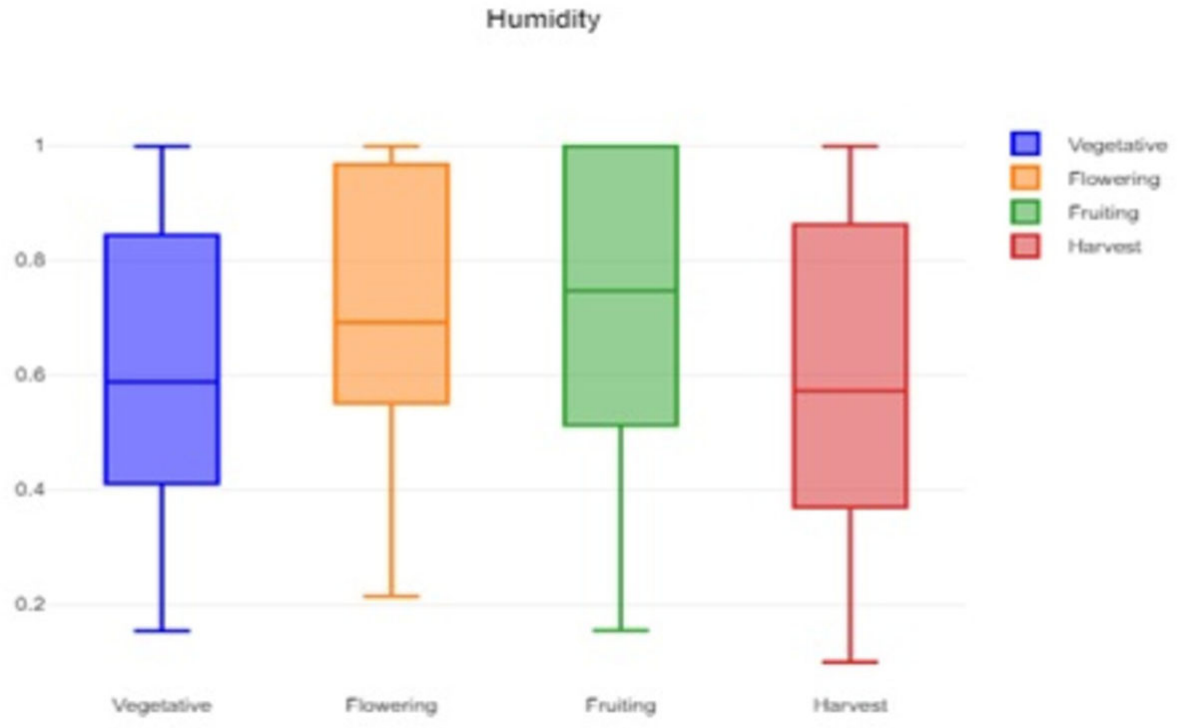
Box plot of the humidity data.

**Figure 3.**
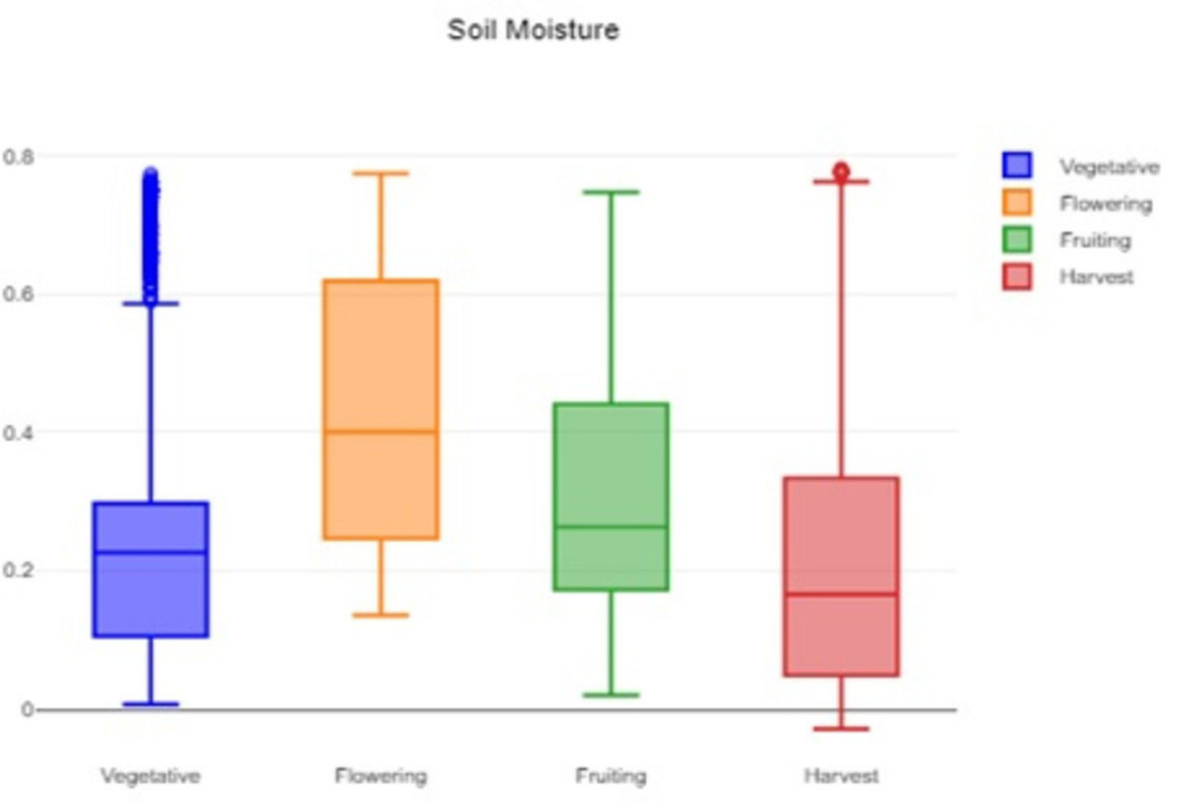
Box plot of the soil moisture data.

**Figure 4.**
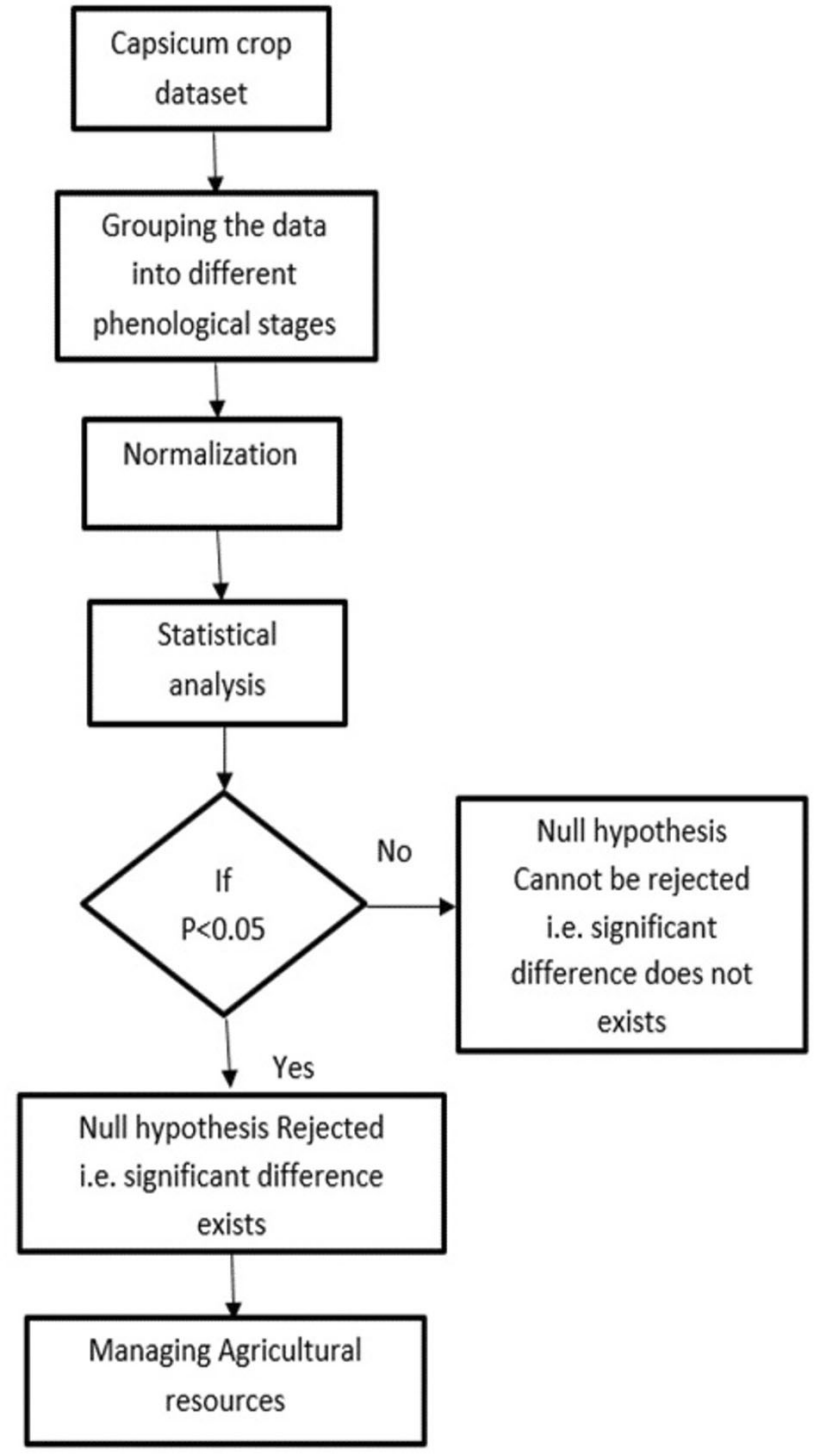
Methodology for the statistical analysis of agricultural parameters.

A box plot of the humidity data is shown in Figure 2, which provides a visual representation of the humidity variability and helps to identify typical, extreme, and unusual conditions. A box plot of the soil moisture data is shown in Figure 3. It helps to assess and understand the soil moisture patterns and identify areas that might require attention or action. The methodology for conducting the statistical analysis of the agricultural parameters of temperature, humidity and soil moisture is shown in Figure 4. Statistical analysis is crucial for understanding and optimizing crop growth and farm management. In this work, the temperature, humidity, and soil moisture dataset of Capsicum was used for statistical analysis. Through surveying and consulting agriculturalists, the Capsicum dataset was divided into four phenological stages. Understanding plant phenology is important for designing a crop management system for adapting to changes in climate and ecological interactions. The min–max normalization technique is used to normalize the data as part of the preprocessing stage.

Statistical hypothesis tests were performed to determine whether there were significant differences or effects related to the parameters. A significance level of α = 0.05 was used to compare the computed p values. A p value of less than 0.05 indicates statistical importance and rejection of the null hypothesis. Statistical analysis is vital in autonomous crop management systems because it enables data-driven decision making and optimization of agricultural resources. Agriculture is increasingly utilizing data for efficient decision-making, enhancing productivity and sustainability. Smart farming is being driven by advancements in data management, utilizing objective information acquired through sensors to minimize resource misuse and environmental pollution. Data-driven agriculture, which involves the incorporation of robotic solutions and artificial intelligence techniques, aims to transform food production to meet population growth needs while saving money.

### Statistical analysis

A new crop variety or production method may significantly boost food production in a particular area. Adaptive research aims to assess a specific innovation’s effectiveness considering regional circumstances. ^28^ With the help of modern agriculture, new methods for increasing farming productivity and efficiency while protecting the environment have been developed. To better comprehend farming tasks and help farmers, agronomists, and experts make decisions, the gathering and examination of enormous agricultural datasets were made possible by current, highly advanced digital equipment and data science.

The use of agricultural technologies, including improved varieties and chemical inputs, is influenced by various variables, such as involvement in organizations, tenure of land, utilization of credit, farmer education, family size, and field size. However, adoption rates differ greatly in terms of geography, technology, and social context. Education in improved varieties may be replaced by extension services, while land tenure encourages natural resource management techniques. Therefore, initiatives to advance agricultural technology in emerging countries must be tailored to local contexts. ^27^ The goal of an autonomous crop management system is to improve its adaptability and usefulness under local conditions. The statistical analysis of agricultural crop parameters is essential for quality control, experimentation, and risk assessment in agriculture. It enables farmers to make informed decisions about resource allocation, risk management, and farming practices to maximize crop yields and profitability. ^29^

A statistical t test was used to analyze the differences between two groups of data. The method compares the mean values of two sets of data and determines if the difference between them is statistically significant. An additional statistical tool, ANOVA, is often known as analysis of variance. ANOVA divides the overall variation in a set of data into two or more elements. All these elements have a particular source of variation attached to it, allowing the analysis to determine how much each of these sources contributed to the overall variation. ANOVA was used most extensively for the analysis of the data obtained from the tests. Both tests are essential in agricultural research for understanding the variation in agricultural elements, including temperature, moisture in the soil, and humidity, and determining the causes that significantly affect these parameters. ^30^ A comparison of the data from different time periods helps to identify the impact of climate change on agricultural parameters and develop crop management systems.

In this work, statistical t tests and ANOVA were performed on the Capsicum crop dataset. Here, the three agricultural parameters of temperature, humidity and soil moisture are considered for the analysis because they are present in plant life cycle events. Understanding the effects of climate change and predicting the impacts of changing phenology on agriculture and resources are important. From the statistical analysis, it is possible to identify the significant differences between the datasets. An autonomous crop management system can be created using this information for the efficient management of agricultural resources.

• **t Test**:

The statistical t test was carried out using the open source statskingdom tool. A t test was applied to compare the means between the different stages of the crop. This study considered agricultural elements, including temperature, humidity, and soil moisture, at various phases to determine whether applying fertilizer or using an irrigation system has a substantial impact on these parameters.

The t-statistic is described by equation (1).

$$t=\frac{\left(\underline{x_{1}}-\underline{x_{2}}\right)-{\left(\mu_{1}-\mu_{2}\right)}^{0}}{\sqrt{\left(\frac{s_{p}^{2}}{n_{1}}+\frac{s_{p}^{2}}{n_{2}}\right)}}$$ (1)

where

*x*_1_- sample means of agricultural parameters during phenological stage I

*x*_2_ - sample mean of agricultural parameters during phenological stage II

( *μ*_1_- *μ*_2_) ^0^ - Hypothesized differences between the means of the two populations at different phenological stages.

*S*_*P*_ - pooled standard deviation

*n*_1 -_ Sample size of agricultural parameters during phenological stage I

*n*_2_ - Agricultural parameter sample sizes during phenological stage II

These are the characteristics of the *t* distribution:

➢ The average is zero.
➢ Around the mean, it is symmetrical.
➢ Between -∞ and ∞ is the variable *t*’s range.
➢ Since there is a distinct distribution for every sample value of *n* - 1, the divisor that is used, the t distribution is a collection of variations used to calculate *s*_2_, also known as degrees of freedom.
➢ The *t* distribution is less centrally peaked and has thicker tails than the normal distribution.
➢ As *n* approaches infinity, the range of *t* approaches the normal distribution.

• **ANOVA test**

The statistical ANOVA test was carried out using an open source statskingdom tool. In this work, the analysis of variance follows a ten-step procedure:

**Description of the data.** The considered dataset was normalized and *classified into different stages of plant phenology.* The classified phonological stages of the Capsicum crop are vegetative, flowering, fruiting, and harvesting. The agricultural crop parameters of temperature, humidity and soil moisture were considered for the analysis.

**Assumptions:** *Independency*: Independent groups and observations that are representative of the populace.

*Normal distribution*: The population was distributed uniformly.

**Hypothesis:** Under the fixed-effects model’s presumptions:

$$H_{0}:\tau_{j}=0,j=1,2,\ldots ,k$$

against the alternative

$$H_{A}:\text{not}\;\mathrm{all}\;\tau_{j}=0$$

**Test statistics:** The evaluation metric is the variance ratio (VR).

**Statistical distribution test:** V.R. has an F distribution when *H*_0_ is true and all the assumptions are satisfied.

**Decision rule:** If the computed value of the test statistic V.R. is greater than or equal to the crucial value of F, the null hypothesis is rejected.

**Test statistic computation:** As indicated in Table 2, in this work, there are two components that contribute to the randomized complete block design: the sum of squares between groups (SSB) and the sum of squares of errors (SSE).

**Table 2.** Randomized complete block design ANOVA table.

Source of Variation	Sum of Squares	Degrees of Freedom	Mean Squares	F Value
Between Groups	SSB= $\sum n_{j}(\bar{X_{j}}-\bar{X)}$ ^2^	Df _1_=k-1	MSB=SSB/(k-1)	F=MSB/MSE
Error	SSE= $\sum \sum (X-\bar{X_{j}})$ ^2^	Df _2_=N-k	MSE=SSE/(N-k)	
Total	SST=SSB+SSE	Df _3_=N-1		

**Statistical decision:** If the calculated F statistic falls within the rejection range, the null hypothesis *H*_0_ is rejected. The alternate hypothesis *H*_*A*_ is accepted, indicating that the variability present in the data is different for all four months.

**Conclusion:** If *H*_0_ is rejected, the alternative hypothesis must be true. If *H*_0_ is not rejected, *H*_0_ might be true.

**p value determination:** For this test, p < 0.05.

The importance of both the t test and ANOVA in agricultural research lies in its ability to help farmers and researchers make data-driven decisions. By conducting both tests, significant differences between crop datasets can be identified. This information can be used for the development of new agricultural technologies for improving agricultural practices.

## Results and Discussion

This study’s main goal was to assess how agricultural factors influence the growth and development of Capsicum plants throughout their phenological stages. To investigate this, the t test was employed, a statistical tool that allows for the comparison of means between two or more groups. The primary objective was to determine whether there are statistically significant differences in these agricultural parameters across various phenological stages of Capsicum crop cultivation. The mean and standard deviation (SD) values of the temperatures during the different phenological stages are shown in Table 3. These statistics provide a quantitative understanding of temperature variations throughout crop growth stages for t tests. The mean and standard deviation (SD) values of humidity during the different phenological stages are displayed in Table 4. This data table provides insights into the humidity levels at each stage.

**Table 3.** The mean and standard deviation scores of the temperatures during the different phenological stages.

Phenological stage	Mean	Standard Deviation (SD)
Vegetative	0.256	0.0653
Flowering	0.263	0.0478
Fruiting	0.2825	0.05796
Harvest	0.3157	0.0630

**Table 4.** The mean and standard deviation scores of humidity during different phenological stages.

Phenological stage	Mean	Standard Deviation (SD)
Vegetative	0.6208	0.254
Flowering	0.7165	0.2192
Fruiting	0.7236	0.252
Harvest	0.596	0.2795

**Figure 5.**
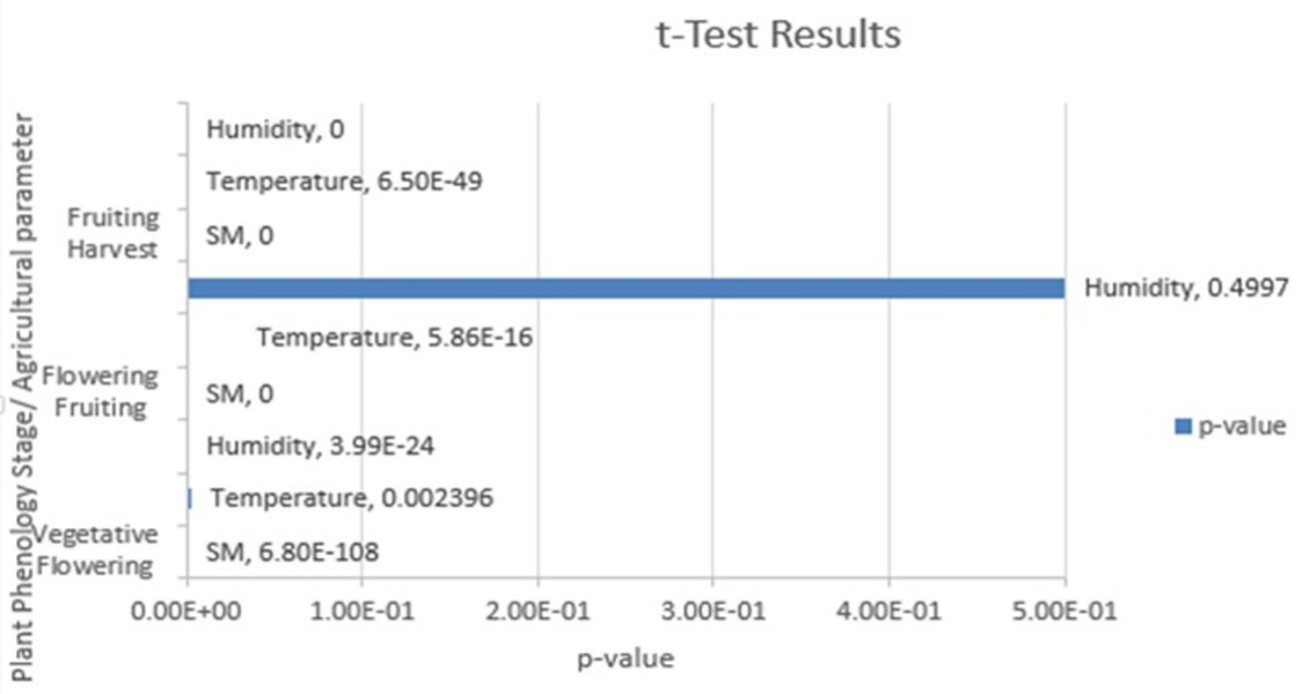
Results of the t test.

The mean and standard deviation (SD) values of soil moisture during the different phenological stages are displayed in Table 5. This information can be valuable for making decisions related to crop management and optimizing irrigation practices. The t test analysis of the agricultural parameters of temperature, humidity, and soil moisture across different phenological stages of Capsicum crops provided compelling evidence to support the rejection of the null hypothesis. The t test results are tabulated in Table 6, and Figure 5 shows the t test results.

**Table 5.** The mean and standard deviation scores of soil moisture during different phenological stages.

Phenological stage	Mean	Standard Deviation (SD)
Vegetative	0.2568	0.1977
Flowering	0.43	0.1985
Fruiting	0.3168	0.201
Harvest	0.2195	0.2143

**Table 6.** Tabulation of t test results.

Phenology stage	Parameter	p value	T statistic	Null hypothesis test
Vegetative-Flowering	Soil moisture	6.801e-108	-22.9061	rejected
	Temperature	0.002396	-3.0385	rejected
	Humidity	3.987e-24	-10.212	rejected
Flowering-Fruiting	Soil moisture	0	12.7624	rejected
	Temperature	5.858e-16	-8.1595	rejected
	Humidity	0.4997	-0.6751	cannot be rejected
Fruiting-Harvest	Soil moisture	0	12.7959	rejected
	Temperature	6.495e-49	-14.9074	rejected
	Humidity	0	12.9944	rejected

With a p value less than the chosen level of significance of 0.05, there were significant differences in the agricultural parameters between the specified phenological stages of the Capsicum crop. These findings underscore the importance of monitoring and managing these parameters throughout the growth cycle of Capsicum plants to optimize their growth and yield. Farmers and researchers can use this valuable insight to make informed decisions and implement targeted strategies for Capsicum cultivation, ultimately contributing to improved agricultural practices and crop productivity.

Analysis of variance (ANOVA) was used to evaluate the variations in temperature, humidity, and soil moisture across the various developmental stages of the Capsicum crop. A significance level (α) of 0.05 was selected as the threshold for determining statistical significance. The ANOVA results are organized and presented in Table 4 for clear reference and interpretation. In this study, we investigated whether there are significant differences in temperature, humidity, and soil moisture levels across different stages of Capsicum crop growth. Table 7 serves as a crucial resource for summarizing the outcomes of the ANOVA. It likely includes key statistical measures such as F values and p values corresponding to each of the three agricultural parameters (temperature, humidity, and soil moisture). These values are essential for making informed decisions regarding the acceptance or rejection of the null hypothesis.

**Table 7.** ANOVA test results.

Parameter	p value	F statistic	Null hypothesis test
Soil moisture	2.22045e-16	274.031881	rejected
Temperature	0	452.388239	rejected
Humidity	1.22125e-15	95.39	rejected

**Figure 6.**
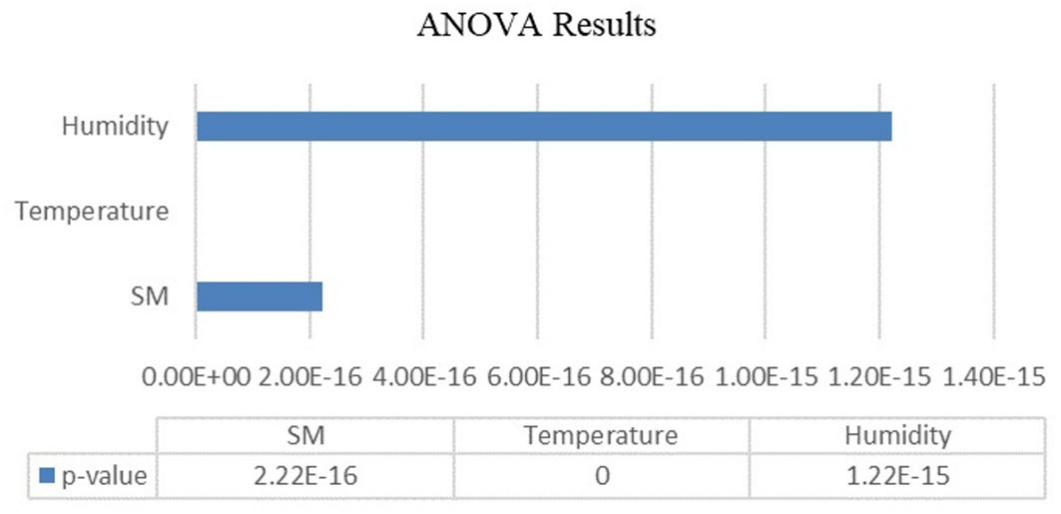
ANOVA results.

The results of ANOVA performed on agricultural parameters for different stages of the Capsicum crop are shown in Figure 6. The obtained p values for soil moisture, temperature and humidity were 2.22045e-16, 0 and 1.22125e-15, respectively. The p value is less than the level of significance (α=0.05). There were significant differences among the groups in terms of these parameters. The use of ANOVA in this context enables agricultural scientists, researchers, and farmers to gain valuable insights into how temperature, humidity, and soil moisture influence the various stages of Capsicum crop development. Such knowledge can inform cultivation strategies, help to optimize environmental conditions and ultimately enhance crop yield and quality.

## Conclusion

The formidable challenge faced by farmers and agriculturalists in analyzing vast amounts of agricultural crop data for resource utilization forecasting has been addressed through the application of open-source statistical tools. The anticipated productivity parameters were identified, contributing to efficient agricultural resource management. Our study specifically scrutinized variations in temperature, humidity, and soil moisture, focusing on Capsicum crop parameters. Through the application of statistical tools such as t tests and ANOVA, significant differences in the parameters were identified across the various phenological stages. The results indicated that temperature, humidity, and soil moisture levels varied notably between stages, underscoring the importance of precise monitoring and management of these factors to optimize crop growth and yield. The use of box plots facilitated the visualization of data variability and outliers, further aiding in decision-making processes.

The statistical findings are instrumental for the development of autonomous crop management systems that can make data-driven decisions. These systems are vital for improving agricultural resource management, enhancing productivity, and promoting sustainability. By leveraging statistical analysis, farmers and researchers can better understand the impacts of climate change and other variables on crop phenology, enabling the adaptation of cultivation practices to changing environmental conditions.

In summary, our research endeavors strive to offer a comprehensive solution to the intricate challenges faced by farmers, providing a pathway to improve crop yield and effective agricultural resource management through the development of an autonomous crop management system.

## Author contributions

Conceptualization – Santhosh KV and Deepashri KM; methodology - Deepashri KM, Santhosh KV and J Satheesh Kumar; software – Deepashri KM and Santhosh KV; validation – Santhosh KV and J Satheesh Kumar; formal analysis – Deepashri KM; investigation, Deepashri KM and Santhosh KV; writing original draft preparation – Deepashri KM and J Satheesh Kumar; writing review and editing – Santhosh KV and J Satheesh Kumar; supervision – Santhosh KV.

## Ethics and consent

Ethics and consent were not required.

## Data Availability

Open Science Framework: Extended data for ‘capsicum crop growth’ is archived at https://doi.org/10.17605/OSF.IO/WJ295. ^25^ This project contains the following underlying data:
• ‘ vegetative_I.csv’ – Sensor data during the process of vegetation • ‘ Flowering_II.csv’ – Sensor data during the process of flowering • ‘ Fruiting_III.csv’ – Sensor data during the process of fruiting • ‘ Harvest_IV.csv’ – Sensor data during the process of harvesting • ‘ ANOVA-test.docx’ – Detail of analysis of data • ‘ Box_plots.docx’ – Results and table of the analysis Data are available under the terms of the Creative Commons Zero “No rights reserved” data waiver (CC0 1.0 Public domain dedication).

## References

[1] Yue Q Guo P Hui W : Towards sustainable circular agriculture: An integrated optimization framework for crop-livestock-biogas-crop recycling system management under uncertainty. *Agric. Syst.* 2022;196:103347. 10.1016/j.agsy.2021.103347

[2] Song C Dong H : Application of Intelligent Recommendation for Agricultural Information: A Systematic Literature Review. *IEEE Access.* 2021;9:153616–153632. 10.1109/ACCESS.2021.3127201

[3] Pantazi X-E Moshou D Bochtis D : *Intelligent data mining and fusion systems in agriculture.* Academic Press;2019. 10.1016/B978-0-12-814391-9.00001-7

[4] Egli DB : *Applied Crop Physiology: Understanding the Fundamentals of Grain Crop Management.* CABI;2021.

[5] Yang X Shu L Chen J : A survey on smart agriculture: Development modes, technologies, and security and privacy challenges. *IEEE/CAA J. Autom. Sin.* 2021;8(2):273–302. 10.1109/JAS.2020.1003536

[6] Gupta VK Parsad R Bhar LM : Statistical Analysis of Agricultural Experiments Part-I: Single Factor Experiments. *ICAR-Indian Agricultural Statistics Research Institute, Library Avenue, Pusa, New Delhi-110.* 2016;12:396.

[7] Junior FM Ribeiro RAC Bianchi RC : Data reduction based on machine learning algorithms for fog computing in IoT smart agriculture. *Biosyst. Eng.* 2022;223:142–158. 10.1016/j.biosystemseng.2021.12.021

[8] Liu G Zhong K Li H : A state of art review on time series forecasting with machine learning for environmental parameters in agricultural greenhouses. *Inf. Process. Agric.* 2024;11(2):143–162. 10.1016/j.inpa.2022.10.005

[9] Xu J Baoxing G Tian G : Review of agricultural IoT technology. *Artif. Intell. Agric.* 2022;6:10–22. 10.1016/j.aiia.2022.01.001

[10] Narwane VS Gunasekaran A Gardas BB : Unlocking adoption challenges of IoT in Indian agricultural and food supply chain. *Smart Agric. Technol.* 2022;2:100035. 10.1016/j.atech.2022.100035

[11] Huat J Aubry C Doré T : Understanding crop management decisions for sustainable vegetable crop protection: a case study of small tomato growers in Mayotte island. *Agroecol. Sustain. Food Syst.* 2014;38(7):764–785. 10.1080/21683565.2014.902895

[12] Majumdar J Naraseeyappa S Ankalaki S : Analysis of agriculture data using data mining techniques: application of big data. *J. Big Data.* 2017;4(1):20. 10.1186/s40537-017-0077-4

[13] Dhaya R Kanthavel R Venusamy K : Data Fusion For Intelligent Decision Making in Agriculture Resource Management. 2021.

[14] Saiz-Rubio V Rovira-Más F : From smart farming towards agriculture 5.0: A review on crop data management. *Agronomy.* 2020;10(2):207. 10.3390/agronomy10020207

[15] Choudhary A : Statistical Analysis of Agriculture Production Performance and Development in Rajasthan. *PalArch’s J. Archaeol. Egypt/Egyptol.* 2020;17(6):12795–12801.

[16] Shah MA Ali AY Qasim H : Statistical analysis of agriculture crop data by two stage systematic sampling. *Am.-Eurasian J. Agric. Environ. Sci.* 2014;14(10):1026–1029.

[17] Gupta R Sharma AK Garg O : WB-CPI: Weather based crop prediction in India using big data analytics. *IEEE Access.* 2021;9:137869–137885. 10.1109/ACCESS.2021.3117247

[18] Wicaksono MG Satrio ES Hendrawan RA : Increasing productivity of rice plants based on IoT (Internet Of Things) to realize Smart Agriculture using System Thinking approach. *Procedia Comput. Sci.* 2022;197:607–616. 10.1016/j.procs.2021.12.179

[19] Chergui N Kechadi MT : Data analytics for crop management: a big data view. *J. Big Data.* 2022;9(1):123. 10.1186/s40537-022-00668-2

[20] Giorgio A Del Buono N Berardi M : Soil moisture sensor information enhanced by statistical methods in a reclaimed water irrigation framework. *Sensors.* 2022;22(20):8062. 10.3390/s22208062 36298410 PMC9610225

[21] Bharadi VA Abhyankar PP Patil RS : Analysis and prediction in agricultural data using data mining techniques. *Int. J. Res. Sci. Eng.* 2017;386–393.

[22] Richard B Qi A Fitt BDL : Control of crop diseases through Integrated Crop Management to deliver climate-smart farming systems for low-and high-input crop production. *Plant Pathol.* 2022;71(1):187–206. 10.1111/ppa.13493

[23] Jaramillo S Graterol E Pulver E : Sustainable transformation of rainfed to irrigated agriculture through water harvesting and smart crop management practices. *Front. Sustain. Food Syst.* 2020;4:437086. 10.3389/fsufs.2020.437086

[24] Gong L Yan J Chen Y : An IoT-based intelligent irrigation system with data fusion and a self-powered wide-area network. *J. Ind. Inf. Integr.* 2022;29:100367. 10.1016/j.jii.2022.100367

[25] Santhosh KV Deepashri KM Kumar S : Capsicum Crop Growth. *OSF.* 2024. 10.17605/OSF.IO/WJ295

[26] Chimdessa A Bekele M Obsa C : Growth and yield response of hot pepper (Capsicum annuum L.) to NPSB blended fertilizers and farm yard manure: a review. *Int. J. Agric. Agribus.* 2019;5(2):88–97.

[27] Ningoji SN Thimmegowda MN Vasanthi BG : Effect of automated sensor-driven irrigation and fertigation on green pepper (Capsicum annuum L.) growth, phenology, quality and production. *Sci. Hortic.* 2024;334:113306. 10.1016/j.scienta.2024.113306

[28] Ruzzante S Labarta R Bilton A : Adoption of agricultural technology in the developing world: A meta-analysis of the empirical literature. *World Dev.* 2021;146:105599. 10.1016/j.dib.2021.107384 PMC8479612 34621923

[29] Ünal Z : Smart farming becomes even smarter with deep learning—a bibliographical analysis. *IEEE Access.* 2020;8:105587–105609. 10.1109/ACCESS.2020.3000175

[30] Daniel WW Cross CL : *Biostatistics: A Foundation for Analysis in the Health Sciences.* Wiley;2018.

