# Supplementary material for: Harnessing Technological Advancements for Enhanced Crop Management: A Study on Capsicum Phenology and Automation in Agriculture
Source: F1000Res. 2025 Sep 9;13:1516. Originally published 2024 Dec 12. [Version 3] doi: 10.12688/f1000research.159518.3 (PMC12828740; doi:10.12688/f1000research.159518.3)
Supplement: Peer review history

# Reviewer report: Reviewer response for version 3

**DOI:** 10.5256/f1000research.187788.r412622

**Published:** 2025-09-25

**Authors:** Mrutyunjay Padhiary

**Affiliations:** 1 Assam University, Silchar, Assam, India

Please add quantified results in the abstract.

 Avoid overuse and rephrase some of the LLM-generated texts.

 Thoroughly proofread and correct minor grammatical errors.

Is the work clearly and accurately presented and does it cite the current literature?

Partly

If applicable, is the statistical analysis and its interpretation appropriate?

Partly

Are all the source data underlying the results available to ensure full reproducibility?

Yes

Is the study design appropriate and is the work technically sound?

Yes

Are the conclusions drawn adequately supported by the results?

Partly

Are sufficient details of methods and analysis provided to allow replication by others?

Yes

Reviewer Expertise:

precision agriculture, farm automation, farm machinery, and AI in farming.

I confirm that I have read this submission and believe that I have an appropriate level of expertise to confirm that it is of an acceptable scientific standard.

## References

[1] : Harnessing AI for Automated Decision-Making in Farm Machinery and Operations. 10.4018/979-8-3693-6230-3.ch008 249-282 10.4018/979-8-3693-6230-3.ch008

# Reviewer report: Reviewer response for version 2

**DOI:** 10.5256/f1000research.184137.r404053

**Published:** 2025-09-04

**Authors:** Janmejay Pant, Dr. Hitesh Kumar Pant

**Affiliations:** 1 Management, kumaun university Nainital, Bhimtal, uttrakhand, India; 2 Graphic Era Hill University, Dehradun, Uttarakhand, India

The article “Harnessing Technological Advancements for Enhanced Crop Management: A Study on Capsicum Phenology and Automation in Agriculture” investigates how environmental parameters (temperature, humidity, and soil moisture) influence Capsicum crops across four phenological stages (vegetative, flowering, fruiting, harvest). Using data collected with Libelium hardware (7,188 samples across four months in 2020), the authors applied statistical methods—t-tests and ANOVA—to identify significant differences across growth stages. The study suggests that these findings can inform autonomous crop management systems, paving the way for improved productivity and sustainability.

 The paper is well-structured and cites current literature extensively, covering IoT, fog computing, data mining, machine learning, and crop-specific studies. It contributes by combining phenology, statistical analysis, and agricultural automation, although several areas need improvement before the article can be considered scientifically strong..

- The article is generally well-written and references 30+ recent studies (2014–2024), ensuring coverage of modern literature.
- However, clarity is weakened by vague terminology in the Abstract and Conclusion (e.g., “substantial variability,” “enhanced productivity”) without quantified results.
- Some sections of the literature review are more detailed (IoT, data mining) while others (climate-smart farming) are underdeveloped.
- Figures are low quality and occasionally missing (e.g., box plots not well interpreted).

- The design (dataset collection, four phenological stages, t-test and ANOVA analysis) is sound and appropriate.
- Use of normalization (min-max scaling) is justified for bounded-input algorithms, though alternative approaches (e.g., z-score) could also have been considered.
- Statistical significance is reported, but no effect size metrics (Cohen’s d, eta-squared) are provided, limiting interpretation of practical significance.
- Dataset limitations (single location, single year) reduce generalizability.

- The statistical methods (t-test, ANOVA) are appropriate and correctly applied.
- Interpretation focuses mainly on statistical significance (p-values) but omits measures of effect size or biological significance.
- Limited discussion of physiological implications (e.g., why soil moisture differs between flowering and harvest, or how this impacts yield).
- The results support the claim that environmental parameters vary significantly across Capsicum growth stages.
- However, broader claims about “enhanced productivity” and “sustainability” are not directly supported by quantified results.
- Biological interpretation and real-world implications (e.g., irrigation or greenhouse automation strategies) are not fully explained.

The article presents a valuable contribution to smart agriculture and crop-specific phenological studies. The study design is sound, the data are openly available, and statistical methods are appropriately applied.

 However, before the article can be considered scientifically strong, the following must be addressed:

1. Quantify results in Abstract and Conclusion; avoid vague terms.
2. Report effect sizes in addition to p-values.
3. Improve figure quality and interpretation (especially box plots).
4. Expand biological/physiological context to explain why variations in temperature, humidity, and soil moisture matter for Capsicum growth.
5. Acknowledge dataset limitations (single location, short time span).

If these issues are addressed, the paper will be significantly stronger and more impactful for the agricultural technology and crop management research community

Is the work clearly and accurately presented and does it cite the current literature?

Partly

If applicable, is the statistical analysis and its interpretation appropriate?

Partly

Are all the source data underlying the results available to ensure full reproducibility?

Yes

Is the study design appropriate and is the work technically sound?

Yes

Are the conclusions drawn adequately supported by the results?

Partly

Are sufficient details of methods and analysis provided to allow replication by others?

Yes

Reviewer Expertise:

Data Analytics and Predictive Modeling (time-series forecasting, statistical analysis, feature engineering).Deep Learning and Soft Computing (neural networks, statistical modeling, intelligent systems).Artificial Intelligence and Machine Learning (with applications in agriculture, healthcare, and anomaly detection).Agricultural Informatics and Smart Farming Systems (application of AI/ML for precision agriculture, crop management, and environmental monitoring).

We confirm that we have read this submission and believe that we have an appropriate level of expertise to confirm that it is of an acceptable scientific standard.

# Reviewer report: Reviewer response for version 2

**DOI:** 10.5256/f1000research.184137.r397680

**Published:** 2025-08-26

**Authors:** Mrutyunjay Padhiary

**Affiliations:** 1 Assam University, Silchar, Assam, India

This study investigates the impact of key agricultural parameters, such as temperature, humidity, and soil moisture, on the phenological stages of Capsicum cultivation. The authors used statistical hypothesis tests, specifically t-tests and ANOVA, to identify significant differences in these parameters across various growth stages of the Capsicum crop. The research highlights the importance of technology, particularly communication and information advancements, for enhancing farming productivity and efficiency and addressing environmental concerns. By collecting and analyzing vast amounts of agricultural data, researchers can strengthen their knowledge about farming practices and make more informed decisions. The study's findings demonstrate substantial variability in these parameters across phenological stages, which points to the importance of tailored crop management strategies. The ultimate goal is to use these insights to develop autonomous crop management systems that can adapt to specific crop needs, improving agricultural productivity and sustainability.

 The statistical analysis and its interpretation are largely appropriate for the stated objectives, demonstrating a sound application of statistical methods to agricultural data. However, there are areas where the interpretation could be strengthened with additional biological context and quantification. The authors should have incorporated biological relevance in revisions and discussed the physiological implications of observed temperature, humidity, and soil moisture variations at each phenological stage.

 Addressing dataset constraints, such as being from a single geographic region or lacking multi-year data, could provide a greater understanding of the generalizability of the findings and the scope of the conclusions.

 Elaborate on the practical implications for autonomous systems, such as how the identified temperature variability during flowering informs the logic of an automated greenhouse ventilation system or how the soil moisture data drives an intelligent irrigation system that adapts to specific phenological needs.

 The study's conclusions are largely supported by the statistical analysis of Capsicum phenology and environmental parameters. The research effectively demonstrates the variability of key parameters across different growth stages and suggests possibilities for autonomous crop management systems based on these findings. The study successfully utilized open-source statistical tools to analyze the Capsicum crop dataset, demonstrating their effectiveness in identifying significant differences and supporting the overall methodology.

 Quantification of impact in conclusions should be addressed, as the initial conclusions were noted as broad and lacking specific quantification of the impact of these variations. The conclusion should directly reference the quantified results to support claims of 'enhanced productivity' or 'optimized crop management.' Understanding why certain parameter changes occur and their direct impact on the plant's growth stages would strengthen the causal link between the results and the conclusions regarding optimized crop management.

 Improve the quality of the figures. Box plots were mentioned but not fully analyzed in terms of their impact on management decisions. Interpreting trends and anomalies from these plots would provide further insights.

Is the work clearly and accurately presented and does it cite the current literature?

Partly

If applicable, is the statistical analysis and its interpretation appropriate?

Partly

Are all the source data underlying the results available to ensure full reproducibility?

Yes

Is the study design appropriate and is the work technically sound?

Yes

Are the conclusions drawn adequately supported by the results?

Partly

Are sufficient details of methods and analysis provided to allow replication by others?

Yes

Reviewer Expertise:

precision agriculture

I confirm that I have read this submission and believe that I have an appropriate level of expertise to confirm that it is of an acceptable scientific standard, however I have significant reservations, as outlined above.

# Response

**Published:** 2025-09-05

**Authors:** Santhosh Venkata

**Affiliations:** Manipal Academy of Higher Education, India

**Reviewer comments:**

 This study investigates the impact of key agricultural parameters, such as temperature, humidity, and soil moisture, on the phenological stages of Capsicum cultivation. The authors used statistical hypothesis tests, specifically t-tests and ANOVA, to identify significant differences in these parameters across various growth stages of the Capsicum crop. The research highlights the importance of technology, particularly communication and information advancements, for enhancing farming productivity and efficiency and addressing environmental concerns. By collecting and analyzing vast amounts of agricultural data, researchers can strengthen their knowledge about farming practices and make more informed decisions. The study's findings demonstrate substantial variability in these parameters across phenological stages, which points to the importance of tailored crop management strategies. The ultimate goal is to use these insights to develop autonomous crop management systems that can adapt to specific crop needs, improving agricultural productivity and sustainability.

**Response:** Thank you for your positive feedback. Your comments affirming the effectiveness of our statistical analysis and the potential of our findings for developing autonomous systems. We will certainly consider your suggestions for future work to further enhance the research’s impact.

 1: The statistical analysis and its interpretation are largely appropriate for the stated objectives, demonstrating a sound application of statistical methods to agricultural data. However, there are areas where the interpretation could be strengthened with additional biological context and quantification. The authors should have incorporated biological relevance in revisions and discussed the physiological implications of observed temperature, humidity, and soil moisture variations at each phenological stage.

**Response: **The specific findings related to biological context and quantification into their statistical interpretations will be integrated in the revised version of the manuscript.

 2: Addressing dataset constraints, such as being from a single geographic region or lacking multi-year data, could provide a greater understanding of the generalizability of the findings and the scope of the conclusions.

**Response: **In future research, the study in diverse geographic regions will be considered to see if the statistical relationships hold and conduct study over multiple consecutive growing seasons to account for inter-annual variability to observe relationships between parameters and phenological stages are consistent over time.

  3: Elaborate on the practical implications for autonomous systems, such as how the identified temperature variability during flowering informs the logic of an automated greenhouse ventilation system or how the soil moisture data drives an intelligent irrigation system that adapts to specific phenological needs.

**Response: **An autonomous crop management system leverages temperature, humidity and soil moisture data to create an optimal environment. The statistical finding that high temperatures during flowering can cause flower drop directly informs the system’s logic. If the temperature approaches a statistically identified stress threshold, the system would automatically activate irrigation and vent mechanisms to prevent flower loss.

 The system’s logic would also adapt to the vegetative stage, where temperature could be kept lower range for robust plant growth. During the fruiting stage, it might allow for slightly higher temperatures as long as the humidity and soil moisture levels are maintained to prevent stress. The system could even made to use historical data to anticipate and adjust its settings, making it intelligent.

 4: The study's conclusions are largely supported by the statistical analysis of Capsicum phenology and environmental parameters. The research effectively demonstrates the variability of key parameters across different growth stages and suggests possibilities for autonomous crop management systems based on these findings. The study successfully utilized open-source statistical tools to analyze the Capsicum crop dataset, demonstrating their effectiveness in identifying significant differences and supporting the overall methodology.

**Response: **Sincere gratitude for providing the positive assessment. The methodology involved the use of open-source statistical tools to analyse crop dataset. The statistical analysis successfully supports about the variability of environmental parameters and the potential for autonomous management systems.

 5: Quantification of impact in conclusions should be addressed, as the initial conclusions were noted as broad and lacking specific quantification of the impact of these variations. The conclusion should directly reference the quantified results to support claims of 'enhanced productivity' or 'optimized crop management.' Understanding why certain parameter changes occur and their direct impact on the plant's growth stages would strengthen the causal link between the results and the conclusions regarding optimized crop management.

**Response: **We have enhanced the quality of the conclusion by incorporating the statistical findings to show the magnitude of the impact as suggested.

 6: Improve the quality of the figures. Box plots were mentioned but not fully analyzed in terms of their impact on management decisions. Interpreting trends and anomalies from these plots would provide further insights.

**Response: **The figures would be replaced with the improved version of figures and  we have incorporate the analysis of the key features of each plot for every phenological stage which would be helpful for interpretation to provide actionable insights for an autonomous system.

# Reviewer report: Reviewer response for version 1

**DOI:** 10.5256/f1000research.175255.r380097

**Published:** 2025-05-28

**Authors:** Dua Weraikat

**Affiliations:** 1 Rochester Institute of Technology-Dubai, Dubai, United Arab Emirates

**Peer-Review Report: "Harnessing Technological Advancements for Enhanced Crop Management: A Study on Capsicum Phenology and Automation in Agriculture"**

 The study addresses a relevant gap in smart agriculture by focusing on Capsicum phenology and the application of statistical tools (t-tests and ANOVA) to analyze environmental parameters across growth stages. While IoT and data-driven methods in agriculture are well-researched, the crop-specific approach and emphasis on phenological variability add novelty. The integration of statistical analysis to inform autonomous crop management systems is timely and aligns with precision agriculture trends.

**Scope:** While the manuscript novelty is in linking Capsicum phenology to statistical analysis for adaptive management, limited discussion of how this work advances beyond existing crop-specific models (e.g., for tomatoes or other crops).

**Abstract:** Terms like "tailored crop management strategies" and "substantial variability" are vague. Quantifying results (e.g., "temperature increased by X°C during harvest") would strengthen impact. Missing explicit mention of **Capsicum** in the results (e.g., "Capsicum exhibited significant differences in soil moisture between stages"). Phrases like "statistical hypothesis tests" and "autonomous crop management systems" assume reader familiarity. A brief contextualization (e.g., "statistical tools to compare growth stages") could improve accessibility. Does not clearly state how this work advances beyond prior studies (e.g., "first statistical analysis of Capsicum phenology for automation").

**Methodology:** The methodology employs a robust dataset (Libellium hardware, 7,188 samples) partitioned into four phenological stages. Statistical tests (t-tests, ANOVA) are appropriate for comparing parameter differences. However, the methodology did not provide the rationale for choosing min-max normalization over alternatives (e.g., z-score). Also, statistical significance (low p-values) is highlighted, but practical significance (e.g., effect size metrics like Cohen’s d) is omitted.

**Literature Review: **The review comprehensively covers IoT, fog computing, and data mining in agriculture, with 30+ references (2014–2024): some sections (e.g., climate-smart farming) are underdeveloped compared to IoT-focused discussions.

**Analysis and Results: **Results demonstrate significant differences (p < 0.05) in temperature, humidity, and soil moisture across phenological stages. However, no discussion of biological relevance (e.g., why temperature increases during harvest) was provided. Also, the box plots are mentioned but not analyzed (e.g., impact on management decisions).

**Conclusion:** Claims like "pathway to improve crop yield" are broad; linking these directly to the study’s findings (e.g., "soil moisture management during flowering could boost yield by X%") would add rigor.

**Missing Components in conclusion** **: **No discussion of dataset constraints (e.g., single geographic region, lack of multi-year data) and Needs specificity (e.g., "testing the proposed system in field trials" or "extending the model to other Solanaceae crops").

**Format and Writing Quality: **The manuscript follows a standard structure (Abstract, Introduction, Methods, Results, etc.), but several issues detract from clarity:

- **Typos**: Inconsistent use of "Capsicum" (e.g., "Cap sicum" on Page 9).
- **Figures/Tables**: Referenced but absent (e.g., Figures 1–3, Table 1), limiting reproducibility assessment.
- **Date anomalies**: Publication dates (Dec 2024) are future-dated, likely a formatting error.
- **Figures Quality**: Figures like Fig 1 and Fig 5 quality are poor and need to be enhanced.

The manuscript presents a well-designed study with significant potential for advancing crop-specific automation in agriculture. Addressing methodological transparency, data visualization, and biological interpretation will strengthen its impact. **Recommend acceptance after minor revisions.**

Is the work clearly and accurately presented and does it cite the current literature?

Partly

If applicable, is the statistical analysis and its interpretation appropriate?

Partly

Are all the source data underlying the results available to ensure full reproducibility?

Partly

Is the study design appropriate and is the work technically sound?

Partly

Are the conclusions drawn adequately supported by the results?

Partly

Are sufficient details of methods and analysis provided to allow replication by others?

Partly

Reviewer Expertise:

Smart Agriculture

I confirm that I have read this submission and believe that I have an appropriate level of expertise to confirm that it is of an acceptable scientific standard, however I have significant reservations, as outlined above.

# Response

**Published:** 2025-06-24

**Authors:** Santhosh Venkata

**Affiliations:** Manipal Academy of Higher Education, India

**Peer-Review Report: "Harnessing Technological Advancements for Enhanced Crop Management: A Study on Capsicum Phenology and Automation in Agriculture"**

 The study addresses a relevant gap in smart agriculture by focusing on Capsicum phenology and the application of statistical tools (t-tests and ANOVA) to analyze environmental parameters across growth stages. While IoT and data-driven methods in agriculture are well-researched, the crop-specific approach and emphasis on phenological variability add novelty. The integration of statistical analysis to inform autonomous crop management systems is timely and aligns with precision agriculture trends.

**Scope:** While the manuscript novelty is in linking Capsicum phenology to statistical analysis for adaptive management, limited discussion of how this work advances beyond existing crop-specific models (e.g., for tomatoes or other crops).

**Response: **As suggested the study on Capsicum phenology now introduces an approach by integrating statistical analysis specifically t-tests and ANOVA to examine environmental parameters across various growth stages. This provides a statistical foundation for adaptive crop management strategies. In contrast, existing models for other crops, such as tomatoes, have also employed statistical analyses to understand phenology and environmental interactions. However, the Capsicum study's emphasis on integrating statistical tools with real-time environmental data and autonomous management systems represents a notable advancement in the field.

**Abstract:** Terms like "tailored crop management strategies" and "substantial variability" are vague. Quantifying results (e.g., "temperature increased by X°C during harvest") would strengthen impact. Missing explicit mention of **Capsicum** in the results (e.g., "Capsicum exhibited significant differences in soil moisture between stages"). Phrases like "statistical hypothesis tests" and "autonomous crop management systems" assume reader familiarity. A brief contextualization (e.g., "statistical tools to compare growth stages") could improve accessibility. Does not clearly state how this work advances beyond prior studies (e.g., "first statistical analysis of Capsicum phenology for automation").

**Response: **Thanks for the suggestion, the results need to be quantified. This work represents a significant step forward in the systematic study of Capsicum phenology. This offers to build a robust framework for monitoring and managing crop development across varying environmental conditions.

**Methodology:** The methodology employs a robust dataset (Libellium hardware, 7,188 samples) partitioned into four phenological stages. Statistical tests (t-tests, ANOVA) are appropriate for comparing parameter differences. However, the methodology did not provide the rationale for choosing min-max normalization over alternatives (e.g., z-score). Also, statistical significance (low p-values) is highlighted, but practical significance (e.g., effect size metrics like Cohen’s d) is omitted.

**Response: **As per the suggestion now the revision is made in the methodology to employ min-max normalization to scale environmental parameters across four Capsicum phenological stages. While this approach is suitable for algorithms requiring bounded input such as fuzzy logic, it may not ne optimal datasets with significant outliners.

 Regarding statistical significance, the study highlights low p-values, indicating that observed differences are unlikely due to chance. However, it is agreed that p-values alone do not measure the magnitude of these differences. Including effect size metrics, such as Cohen's d, would provide additional context by quantifying the size of the differences between groups will be considered.

**Literature Review: **The review comprehensively covers IoT, fog computing, and data mining in agriculture, with 30+ references (2014–2024): some sections (e.g., climate-smart farming) are underdeveloped compared to IoT-focused discussions.

**Response: **Thanks for providing the feed back, we have not tried to review on the climate-smart farming section will be expanded to provide a more balanced perspective on the role of IoT in modern agriculture, addressing both technological advancements and sustainable practices.

**Analysis and Results: **Results demonstrate significant differences (p < 0.05) in temperature, humidity, and soil moisture across phenological stages. However, no discussion of biological relevance (e.g., why temperature increases during harvest) was provided. Also, the box plots are mentioned but not analyzed (e.g., impact on management decisions).

**Response:**

 The observed environmental variations impact Capsicum physiology and development and their biological relevance will be incorporated in the manuscript.

 An interpretation of the box plots, highlighting trends and anomalies across phenological stages will be discussed.

**Conclusion:** Claims like "pathway to improve crop yield" are broad; linking these directly to the study’s findings (e.g., "soil moisture management during flowering could boost yield by X%") would add rigor.

**Response: **Such specific findings will be incorporated in the manuscript to provide more precise recommendations for irrigation practices, thereby enhancing its practical relevance for farmers.

**Missing Components in conclusion: **No discussion of dataset constraints (e.g., single geographic region, lack of multi-year data) and Needs specificity (e.g., "testing the proposed system in field trials" or "extending the model to other Solanaceae crops").

**Response: **The missing components in conclusion have been addressed. Further the study can provide more comprehensive and actionable insights into Capsicum phenology, contributing to improved agricultural practices and crop management strategies.

**Format and Writing Quality: **The manuscript follows a standard structure (Abstract, Introduction, Methods, Results, etc.), but several issues detract from clarity:

 Typos: Inconsistent use of "Capsicum" (e.g., "Cap sicum" on Page 9).

 Figures/Tables: Referenced but absent (e.g., Figures 1–3, Table 1), limiting reproducibility assessment.

 Date anomalies: Publication dates (Dec 2024) are future-dated, likely a formatting error.

 Figures Quality: Figures like Fig 1 and Fig 5 quality are poor and need to be enhanced.

**Response: **The following recommendations will be taken and suggestions will be incorporated in the manuscript.
